# Regulating the T7 RNA polymerase expression in *E. coli* BL21 (DE3) to provide more host options for recombinant protein production

**DOI:** 10.1186/s12934-021-01680-6

**Published:** 2021-09-26

**Authors:** Fei Du, Yun-Qi Liu, Ying-Shuang Xu, Zi-Jia Li, Yu-Zhou Wang, Zi-Xu Zhang, Xiao-Man Sun

**Affiliations:** 1 School of Food Science and Pharmaceutical Engineering, Nanjing Normal University, 2 Xuelin Road, Qixia District, Nanjing, People’s Republic of China; 2 Nanjing Foreign Language School, Nanjing, People’s Republic of China

**Keywords:** BL21 (DE3), Inducible promoter, T7 RNA polymerase, Recombinant protein

## Abstract

*Escherichia coli* is the most widely used bacterium in prokaryotic expression system for the production of recombinant proteins. In BL21 (DE3), the gene encoding the T7 RNA polymerase (T7 RNAP) is under control of the strong lacUV5 promoter (P_lacUV5_), which is leakier and more active than wild-type lac promoter (P_lacWT_) under certain growth conditions. These characteristics are not advantageous for the production of those recombinant proteins with toxic or growth-burdened. On the one hand, leakage expression of T7 RNAP leads to rapid production of target proteins under non-inducing period, which sucks resources away from cellular growth. Moreover, in non-inducing or inducing period, high expression of T7 RNAP production leads to the high-production of hard-to-express proteins, which may all lead to loss of the expression plasmid or the occurrence of mutations in the expressed gene. Therefore, more BL21 (DE3)-derived variant strains with rigorous expression and different expression level of T7 RNAP should be developed. Hence, we replaced P_lacUV5_ with other inducible promoters respectively, including arabinose promoter (P_araBAD_), rhamnose promoter (P_rhaBAD_), tetracycline promoter (P_tet_), in order to optimize the production of recombinant protein by regulating the transcription level and the leakage level of T7 RNAP. Compared with BL21 (DE3), the constructed engineered strains had higher sensitivity to inducers, among which rhamnose and tetracycline promoters had the lowest leakage ability. In the production of glucose dehydrogenase (GDH), a protein that causes host autolysis, the engineered strain BL21 (DE3::ara) exhibited higher biomass, cell survival rate and foreign protein expression level than that of BL21 (DE3). In addition, these engineered strains had been successfully applied to improve the production of membrane proteins, including *E. coli* cytosine transporter protein (CodB), the *E. coli* membrane protein insertase/foldase (YidC), and the *E. coli* F-ATPase subunit b (Ecb). The engineered strains constructed in this paper provided more host choices for the production of recombinant proteins.

**Supplementary Information:**

The online version contains supplementary material available at 10.1186/s12934-021-01680-6.

## Introduction

*Escherichia coli* BL21 (DE3) and pET expression system are the most representative recombinant protein expression systems [1]. In BL21 (DE3), expression of the gene encoding the target protein, which is on the pET plasmid, is driven by the chromosomally encoded bacteriophage T7 RNA polymerase (T7 RNAP). The T7 RNAP specifically recognizes the T7 promoter and transcribes eight times faster than *E. coli* RNAP [2–4]. The gene encoding the T7 RNAP is governed by the isopropyl β-d-1-thiogalactopyranoside (IPTG)-inducible lacUV5 promoter (P_lacUV5_), which is a strong variant of the wild-type lac promoter (P_lacWT_) [5]. The reasoning behind the choice of these components to create a protein overexpression system is straight-forward, the more mRNA is synthesized, the more protein can be produced. However, this system is not suitable for those recombinant proteins toxic or growth-burdened.

The ideal production of recombinant proteins by BL21 (DE3) includes two periods, where biomass is accumulated rapidly during non-inducing period, while protein production is achieved during the inducing period. Moreover, the expression level of T7 RNAP is important for the production of recombinant proteins [6]. For easy-to-express proteins, production of proteins in BL21 (DE3) is often limited because a large portion of feedstock will be used to produce biomass [7, 8]. To address this issue, many strategies were developed to regulate the allocation of resources between cell growth and protein production. A gene growth switch based on CRISPRi was developed in *E. coli* to increase the expression of T7 RNAP by targeting genes related to cell growth and DNA replication, then the GFP-protein production was increased by 2.2-fold [9]. Recently, Stargardt et al. realized the decoupling of cell growth and protein expression by introducing the phage T7 gp2 gene (an inhibitor of *E. coli* RNAP), and the most of the resources in the cell flowed to the expression of T7 RNAP to promote the target protein production when there was enough biomass [10, 11]. However, for hard-to-express proteins, high-expression of target proteins tends to overwhelm host cell. To address this issue, a series of BL21 (DE3)-derived variant strains had been developed such as C41 (DE3), C43 (DE3), and Mutant56 (DE3) [12, 13]. It is worth mentioning that the rationale behind these variant strains is that the expression level of the T7 RNAP was reduced, which in turn produced less T7 RNAP corresponding to the target recombinant protein. For example, Sun et al. constructed a non-autolytic strain capable of efficiently producing recombinant proteins by hybridizing the weak lac promoter with the strong P_lacUV5_, which downregulated expression of T7 RNAP to realize the efficient production of enzymes [14].

In addition, the P_lacUV5_ mutant is CRP-independence, which is leakier than P_lac_ [15]. If there is more T7 RNAP expression in the non-inducing period, the cells will begin to produce protein before they have accumulated a certain amount of biomass. The higher basic leakage expression of P_lacUV5_ in the absence of inducer, which causes toxicity to cells and leads to loss of the expression plasmid or the occurrence of mutations in the expressed gene [16, 17]. Therefore, rigorous expression of T7 RNAP is important for the stability of protein production systems. The lacI mutant was developed, which only can bind to the lacO (lac operator) and significantly reduced the basic leakage expression of P_lacUV5_ [18]. De Gier developed a system termed Lemo21 (DE3), where the transcriptional activity of the T7 RNAP was controlled by the cellular abundance of its inhibitor T7 lysozyme, whose expression was in turn placed under the tight control of the P_rhaBAD_, which controlled the activity of T7 RNAP and overcame the problem of leaky expression in T7 RNAP-based protein expression systems and showed improved target protein yields, especially for membrane proteins [1]. Furthermore, there is a disadvantage that the addition of the inducer IPTG will cause chemical toxicity to the cells [16, 17]. To eliminate the toxicity of IPTG to cells, the BL21-AI < gp2 > strain using arabinose promoter to drive transcription of the T7 RNAP was developed and used to successfully overexpress toxic proteins [11]. Based on these examples it becomes evident that an optimal and rigorous expression level of T7 RNAP is important to achieve maximal recombinant protein yield.

In this study, we constructed three BL21 (DE3)-derived variant strains with different transcription level and leakage expression of T7 RNAP, which expands host strains for recombinant protein expression. Specifically, engineered strains were constructed by replacing P_lacUV5_ with other inducible promoters: arabinose promoter (P_araBAD_), rhamnose promoter (P_rhaBAD_), tetracycline promoter (P_tet_). Furthermore, the novel engineered strains were successfully applied to overproduce one autolytic protein and three membrane proteins, glucose dehydrogenase (GDH), *E. coli* cytosine transporter protein (CodB), the *E. coli* membrane protein insertase/foldase (YidC) and the *E. coli* F-ATPase subunit b (Ecb), which had been reported to be difficult to produce in *E. coli* strains [12, 14, 16, 19]. This article has important disquisitive significance for improving the expression of recombinant protein.

## Materials and methods

### Bacterial strains and plasmid constructions

All plasmids and recombinant proteins used in this study are listed in Additional file 1: Table S1, and the DNA primers are listed in Additional file 1: Table S2. The *E. coli* DH5α was used for plasmid construction, and BL21 (DE3) was applied for gene expression. All plasmids used to express recombinant protein are derived from pET24. The fragments plasmid skeleton obtained by PCR were linked by Gibson assembly. In the process of plasmid construction, the recombinant proteins expressed in cells was C-terminally fused to EGFP. The constructed plasmid was transformed into DH5a by chemical transformation method and the transformants were confirmed by colony PCR. Correct colonies were screened and plasmids were obtained by plasmid extraction kit.

### Culture conditions

All *E. coli* strains were cultivated in Luria Bertani (LB) medium which contained 10 g/L tryptone, 5 g/L yeast extract and 10 g/L NaCl at 37 °C with constant shaking at 220 rpm, and added 2% agar if it was solid LB medium. The fermentation strains grown in Terrific Broth (TB) mediumat 28 °C with constant shaking at 220 rpm. The medium was supplemented with 50 µg/mL kanamycin (kan) or spectinomycin (spec) according to the screening markers carried by intracellular plasmids.

Flask fermentation was performed as follows: the newly constructed strains with different promoters harboring recombinant protein plasmids were grown in 3 mL of LB liquid medium overnight at 37 °C. Then, 300 µL of the resulting culture were used to inoculate 30 mL of TB medium in a 250 mL shake flask. Cells were cultured at 37 °C to an OD_600_ of 2–4, at which point Isopropyl β-d‐1‐thiogalactopyranoside (IPTG), l-arabinose, rhamnose (Rha) or anhydrotetracycline (aTc) was added to a final concentration of 0.3 mM, 10 mM, 10 mM, 2.4 µM, respectively. The fermentation was allowed to continue at 28 °C for an additional 60 h. The fermentation conditions used in this study were optimized [20].

### Construction of engineered strains

In this paper, the four inducible promoters used included P_lacUV5_, P_araBAD,_ P_rhaBAD_ and P_tet_. Plasmids pTarget-ara/pTarget-rha/pTarget-tet were obtained by placing different promoter sequences on gRNA plasmids respectively and the template DNA with 1,000 bp homologous arms was prepared by PCR. The primers of these promoters obtained by PCR were listed in Additional file 1: Table S2. We used the modified CRISPR/Cas9 system ( pEcCas/pEcgRNA) to edit the genome of *E. coli* BL21 (DE3) [21]. The CRISPR/Cas9 system contains two plasmids. The first plasmid, pEcCas, was transformed into BL21 (DE3) strain. Then the pEcgRNA plasmid with donor fragment was transferred into the above strain by electroporation (1.85 kV, 200 Ohm, 25 µF), then recovered for 1 h in 1 mL LB medium at 37 °C. The extent of promoter (i.e., 749,956–751,219 bp) controlling T7 RNAP of BL21 (DE3) was replaced separately by other inducible promoters and their auxiliary expression proteins. The mutants were confirmed by colony PCR and gene sequencing. Correct colonies were then screened and further subjected to the plasmid curing process to get empty engineered strains [21].

### Scanning fluorescence microscope and fluorescence intensity

The pET24a-EGFP plasmid was constructed by amplifying the EGFP fragment with primers EGFP-F and EGFP-R and cloning into pET24a, which was digested with Dph1 in advance. Plasmids were respectively transferred into newly constructed strains and original strains BL21 (DE3). To prepare competent cells, a transformant cultivated at 37 °C until the OD_600_ reached 0.6–0.8. The calcium chloride method was used to transfer the pET24a-EGFP plasmid into the host strain, followed by recovery at 37 °C for 1 h. Then, positive colonies were screened on LB agar plate with 50 µg/mL kan at 37 °C. One colony was randomly selected and pre-cultured in LB with antibiotic at 37 °C. Then, 300 µL of the resulting culture were used to inoculate 30 mL of TB medium in a 250 mL shake flask. The recombinant protein expression strains were cultured according to the fermentation method we described above to express the target protein at 28 ℃ for 18 h.

The cell growth was monitored by absorbance at 600 nm and a small amount of cells were observed under fluorescence microscope. 500 µL of cells was centrifuged at 12,000 rpm for 5 min and then resuspended in 500 µL of phosphate-buffered saline (PBS). One hundred microliters of the resuspensions were transferred to 96-well Black Optiplate 96 F plates. The whole cell fluorescence was detected with a 485 nm excitation wavelength and 535 nm emission wavelength. All experiments were performed in triplicates.

### Plasmid stability analysis

Samples were taken at 24, 36 and 48 h of fermentation, and diluted to the same OD using PBS. 100 µL of dilution liquid was coated on LB agar plate with 50 µg/mL kanamycin. After culturing for 12 h, 100 single colonies were picked randomly and spotted on LB agar plate and LB agar plate containing 50 µg/mL kan respectively. After cultivation for 12 h at 37 °C, colonies growing on LB agar plate but not on LB with 50 µg/mL kan were interpreted that the plasmid has been lost. The plasmid stability was calculated as the number of colonies with growth in kanamycin medium/100 × 100%.

### Protein expression based on engineered strains

The recombinant proteins expressed in four strains was C-terminally fused to EGFP. Therefore, the expression level of recombinant protein can be observed by fluorescence intensity. The engineered strains with different recombinant protein plasmids were cultivated in 5 mL of LB liquid medium overnight at 37 °C. Then, 300 µL of the resulting culture were used to inoculate 30 mL of TB medium in a 250 mL shake flask. The inducers were added as described above when OD was 2–4 and the fermentation was allowed to continue at 28 °C to express protein. The processed samples of fermentation were examined according to the method described above to calculate unit cell fluorescence value and plasmid stability. All experiments were performed in triplicates.

## Results

### Construction and characterization of engineered strains

**Fig. 1:**
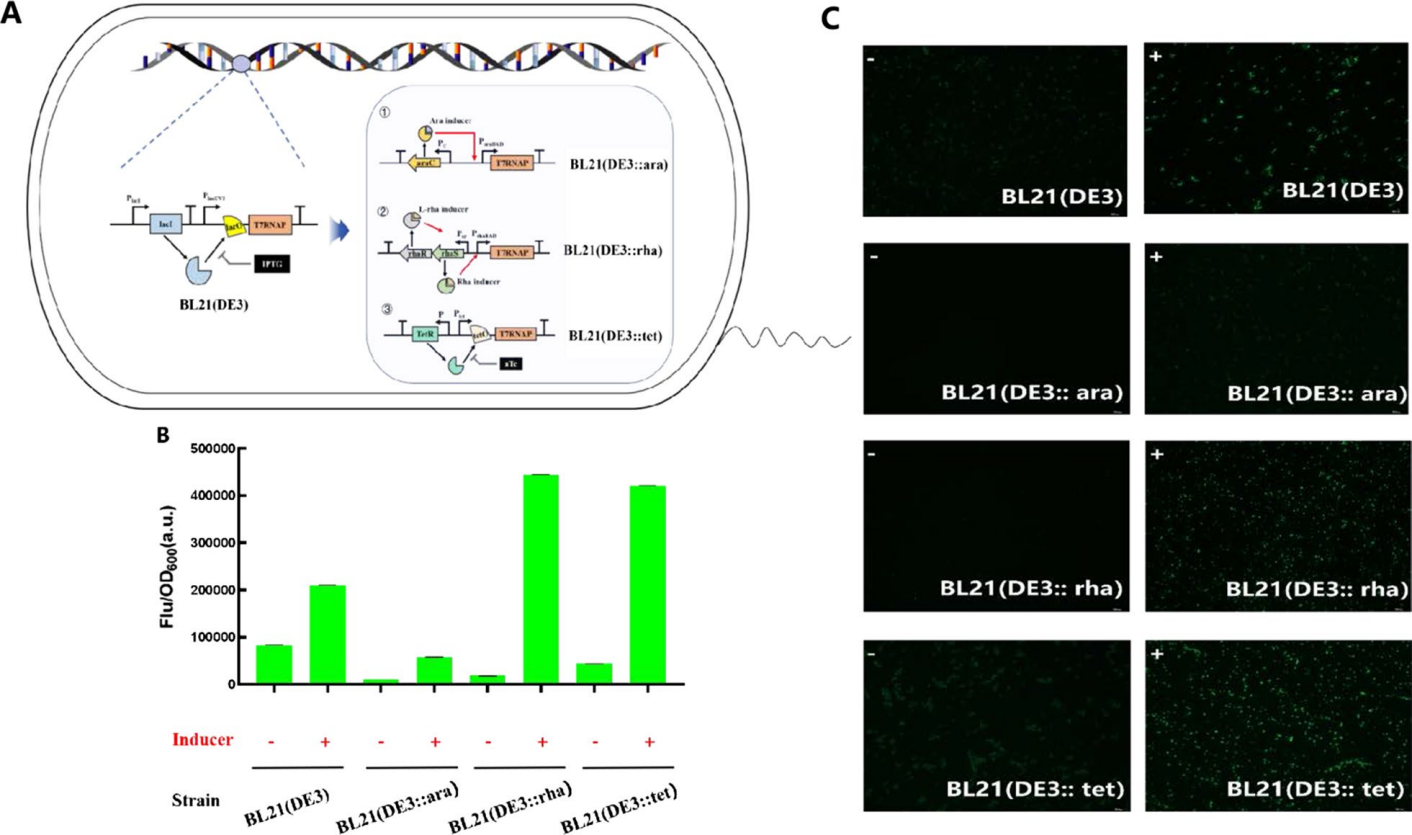
Construction of three engineered strains and fluorescence intensity of EGFP fermentation for 18 h. **A** Genetic design of engineered T7 RNAP in *E. coli* strains BL21 (DE3). Three engineered T7 RNAP expression strains which containing different inducible promoters were constructed. **B** Fluorescence intensity of different strains containing EGFP plasmid with or without inducer and fluorescence diagram (**C**). The error bar denotes the standard deviation of the mean from the three replicates

The constructed DNA expression cassettes of different promoters were integrated into the chromosome of *E. coli* BL21 (DE3) by CRISPR/Cas9 system [21]. And the engineered strains were respectively denoted as BL21 (DE3::ara), BL21 (DE3::rha), BL21 (DE3::tet) (Fig. 1a). Firstly, the leakage ability of the inducible promoters and the transcription level of T7 RNAP of the engineered strains were characterized by fluorescence intensity. As shown in Fig. 1b, under the condition of no inducer, there was obviously higher florescence intensity in original strain BL21 (DE3), which proved that the P_lacUV5_ has a high basic leakage expression. However, it can be seen that the P_rhaBAD_ and the P_tet_ exhibited the lowest leakage ability. When relative inducer was added, the average fluorescence value of BL21 (DE3::rha) and BL21 (DE3::tet) reached 430,000 a.u, which was 2.11 and 2.0 times higher than that of BL21 (DE3), respectively. In BL21 (DE3::rha), the fluorescence intensity in the existence of inducer was 24.26 times higher than that of none inducer, while that of BL21 (DE3) strain was 2.52 times as high as that of none inducer. The same phenomenon can also be obtained in Fig. 1c. Compared with the engineered strains, the fluorescence image of strain BL21 (DE3) was the brightest without induction. After induction, the brightness of the fluorescent images of strains BL21 (DE3::rha) and BL21 (DE3::tet) were higher than that of BL21 (DE3) (Fig. 1c). In general, the P_rhaBAD_ was considered to be the best rigorously promoter, as it had the lowest leakage expression.

### Application of engineered host strains in GDH production

**Fig. 2:**
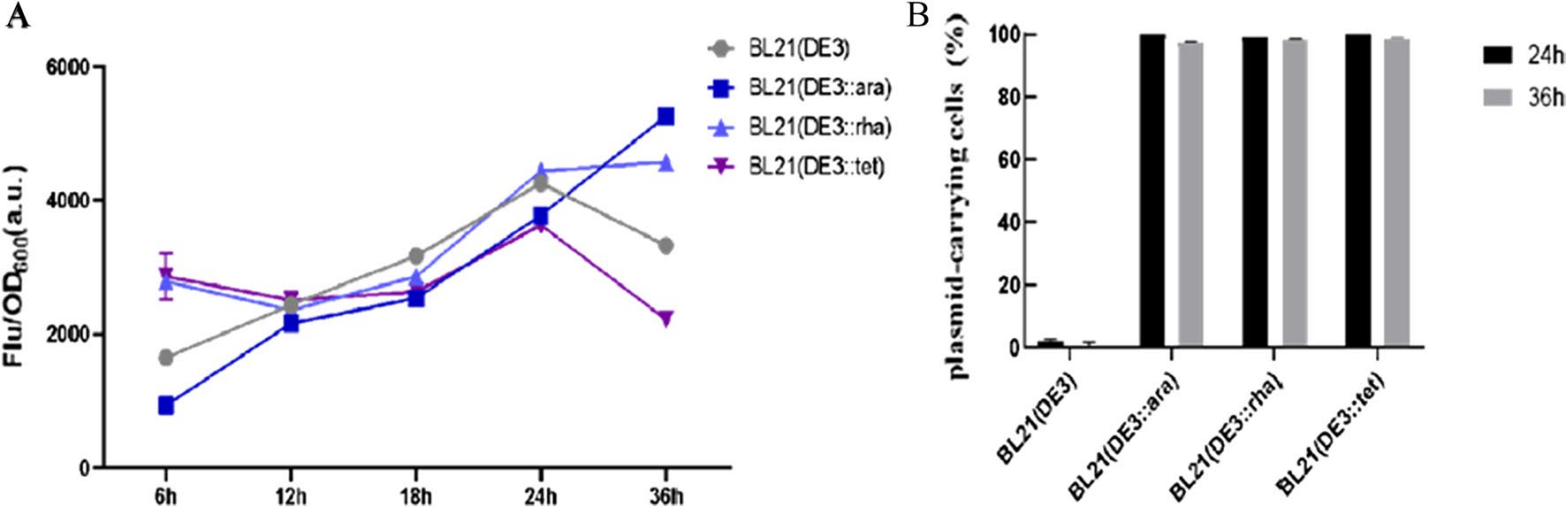
Expression of GDH-EGFP in different strains. **A** Fluorescence intensity of GDH-EGFP in the whole fermentation process. **B** The percentage of plasmid-carrying cells of BL21 (DE3), BL21 (DE3::ara), BL21 (DE3::rha) and BL21 (DE3::tet) was tested during the entire fermentation period. Values and error bars represent the means and the deviations from triplicate experiments. *GDH* glucose dehydrogenase

Studies had shown that when BL21 (DE3) overexpressed glucose dehydrogenase (GDH), severe cell autolysis was induced, resulting in lower protein production [14]. Therefore, we investigated whether the three engineered strains can improve the expression of GDH (Fig. 2a). In the first 24 h of GDH production, the growth status and protein production of strain BL21 (DE3) were optimal. However, the growth of the BL21 (DE3) slowed down and the biomass decreased drastically, while the cell biomass and the fluorescence intensity of the three engineered strains continued to rise from 24 to 36 h. In particular, at the 36 h, the fluorescence intensity of strain BL21 (DE3::ara) and BL21 (DE3::rha) was 1.57 and 1.37 times as high as that of BL21 (DE3), respectively.

Furthermore, the intracellular stability of the expression plasmid GDH at the later stage of fermentation was tested (Fig. 2b). As shown in Fig. 2b, the ratio of plasmid-carrying cells in BL21 (DE3) continued to decline with increasing fermentation time, reaching a low of 2.17% at 36 h. By contrast, the ratio of plasmid-carrying cells in BL21 (DE3::ara), BL21 (DE3::rha) and BL21 (DE3::tet) remained about 100%, 99% and 100% at 36 h, respectively. The plasmid stability of the engineered host strains were much higher than that of the parent strain BL21 (DE3). It indicated that downregulating the expression level of T7 RNAP and reducing the leakage expression of promoter can improve the stability of plasmid, reduce the mutation of expressed gene and promote protein expression. Among them, the engineered strain BL21 (DE3::ara) was more effective for producing GDH, which was resistant to autolysis by downregulating the T7 RNAP expression [14]. In general, the survival rate of the three engineered strains were still high, and the expression plasmid existed stably in the cells at 36 h, which indicated that the yield of autolytic proteins can be further improved by prolonging the fermentation time.

### Application of engineered host strains in membrane proteins production

**Fig. 3:**
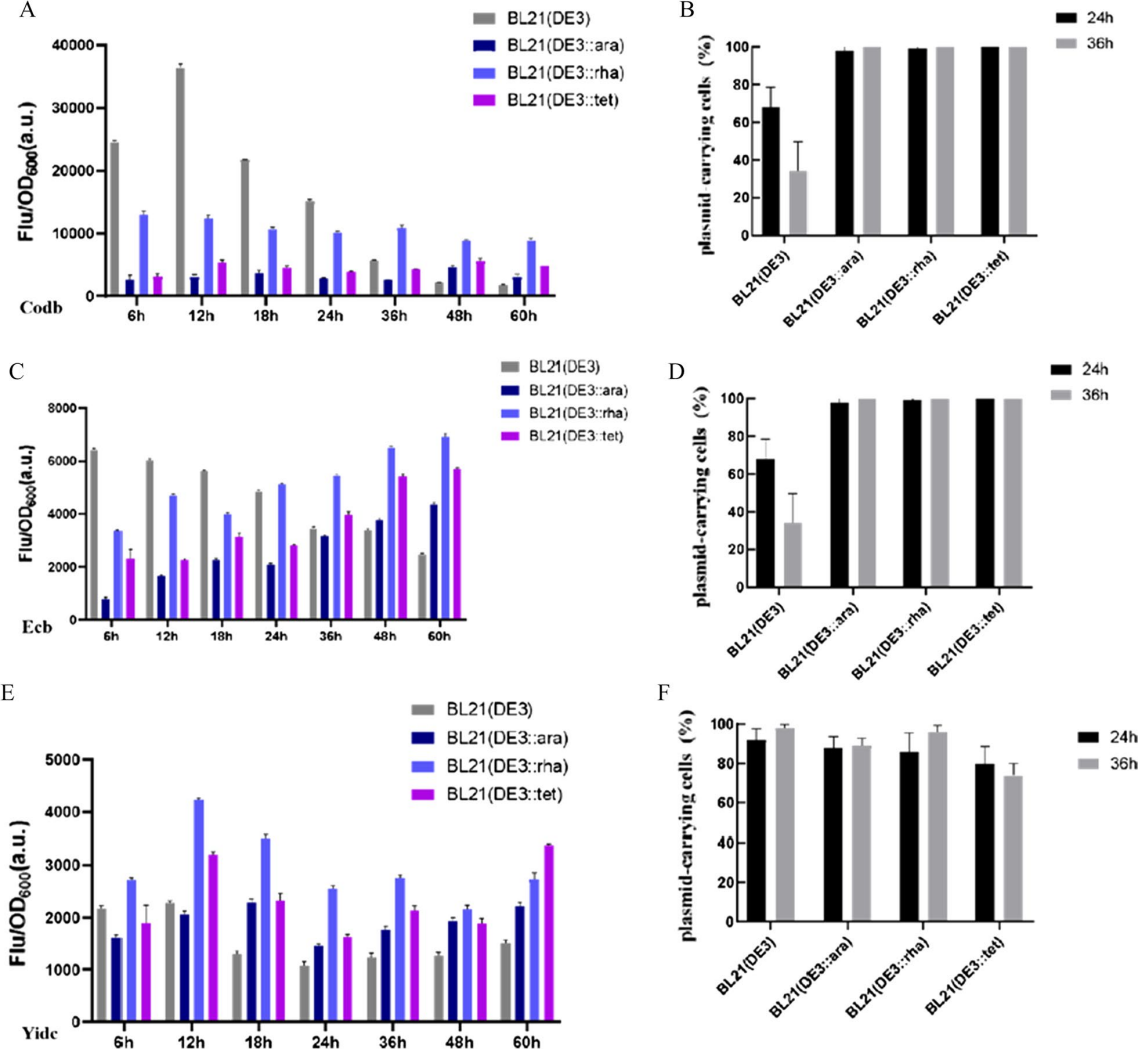
Expression of three membrane proteins in three engineered strains and control strain BL21 (DE3). **A** Fluorescence intensity of Codb-EGFP of BL21 (DE3), BL21 (DE3::ara), BL21 (DE3::rha) and BL21 (DE3::tet) in the whole fermentation process and the percentage of plasmid-carrying cells in **B**. **C** Fluorescence intensity of Ecb-EGFP of BL21 (DE3), BL21 (DE3::ara), BL21 (DE3::rha), and BL21 (DE3::tet) in the whole fermentation process and the percentage of plasmid-carrying cells in **D**. **E** Fluorescence intensity of Yidc-EGFP of BL21 (DE3), BL21 (DE3::ara), BL21 (DE3::rha), and BL21 (DE3::tet) in the whole fermentation process and the percentage of plasmid-carrying cells in **F**. Values and error bars represent the means and the deviations from triplicate experiments. CodB, *E. coli* cytosine transporter protein. Ecb, *E. coli* F-ATPase subunit b. YidC, *E. coli* membrane protein insertase/foldase

The application of engineered strains in the expression of three membrane proteins (Codb, Ecb, and Yidc) was further investigated. For each of these membrane proteins, we carefully chose *E. coli* host strains based on previous attempts to express these proteins. For example, CodB was hardly produced in *E. coli* K-12 [19], and most of the host cells died when Ecb and Yidc were expressed in BL21 (DE3) [18]. The results of three membrane proteins production were shown in Fig. 3. In the production of membrane protein Codb, after adding the inducer, the fluorescence intensity of strain BL21 (DE3) reached 36,461 a.u at 12 h, which were 11.8, 3.0 and 6.8 times higher than those of other three engineered strains at the same stage, respectively (Fig. 3a). It was no doubt that strain BL21 (DE3) had great advantages in producing membrane protein Codb before 12 h. However, the fluorescence intensity of the strain BL21 (DE3) dropped sharply, while the fluorescence intensity of the three engineered strains continued to increase steadily after 24 h. Among them, the fluorescence intensity of the strain BL21 (DE3::rha) reached 10,870 a.u., while cell biomass has little change, and the average ratio of plasmid-carrying cells was about 98% (Fig. 3b). Even at 60 h, the fluorescence intensity of the strain BL21 (DE3::rha) was the highest, which was 2.7 times as high as that of BL21 (DE3). So the strain BL21 (DE3::rha) was considered to be the more suitable host strain for Codb production if the production of membrane protein was increased by prolonging the fermentation time. Similarly, the fluorescence intensity of strain BL21 (DE3) firstly increased and then decreased for the production of Ecb, while the three constructed strains had been showing an upward trend (Fig. 3c). The fluorescence intensity of strain BL21 (DE3) was the highest (i.e., 6410 a.u.) at 18 h, but there was no significant difference between strain BL21 (DE3::rha) and BL21 (DE3) at 24 h. At 60 h, the fluorescence of BL21 (DE3::rha) and BL21 (DE3::tet) were 6913 a.u. and 5711 a.u., which were 2.3 and 1.6 times higher than those of strains BL21 (DE3), respectively. The ratio of plasmid-carrying cells in three engineered strains remained about 98% at 48 h (Fig. 3d). So BL21 (DE3::rha) was considered to be the better host choice for Ecb efficient production by prolonging the fermentation time, as it downregulated the expression level of T7 RNAP and improved the stability of plasmid. In the production of Yidc, the fluorescence intensity of BL21 (DE3::tet) reached 3519 a.u. at 18 h, which was 2.7 times that of the BL21 (DE3) (Fig. 3e), and 96% of the plasmids were stably existed (Fig. 3f). The fluorescence intensity of the strain BL21 (DE3::tet) was the highest even at 60 h. The strain BL21 (DE3::tet) was more suitable for Yidc production. In short, the best host for producing different membrane proteins was different, which was contributed to requirements for T7 RNAP of different membrane proteins. Therefore, the three engineered strains had been successfully applied to the production of membrane proteins, and three engineered strains can further improve the membrane proteins yield by regulating the rigorous expression of T7 RNAP and the leakage level of promoter.

### Fine regulation of the concentration of inducer can further improve target protein production

It had been reported that the concentration of the inducer has a great influence on the final expression yield of the protein [18, 22]. In order to further improve the application potential of the engineered strains, the production capacity of the three engineered strains and strain BL21 (DE3) were investigated by adding different inducer concentrations on the basis of the previous research. The addition concentration of IPTG was 0.1 mM, 0.3 mM and 0.5 mM, respectively. The concentration of L-arabinose was 2.5 mM, 10.0 mM, 20.0 mM, respectively. The concentration of rhamnose (Rha) added was 2.0 mM, 10.0 mM, 40.0 mM, respectively. And anhydrotetracycline (aTc) was 0.6 µM, 2.4 µM, 7.0 µM, respectively.

**Fig. 4:**
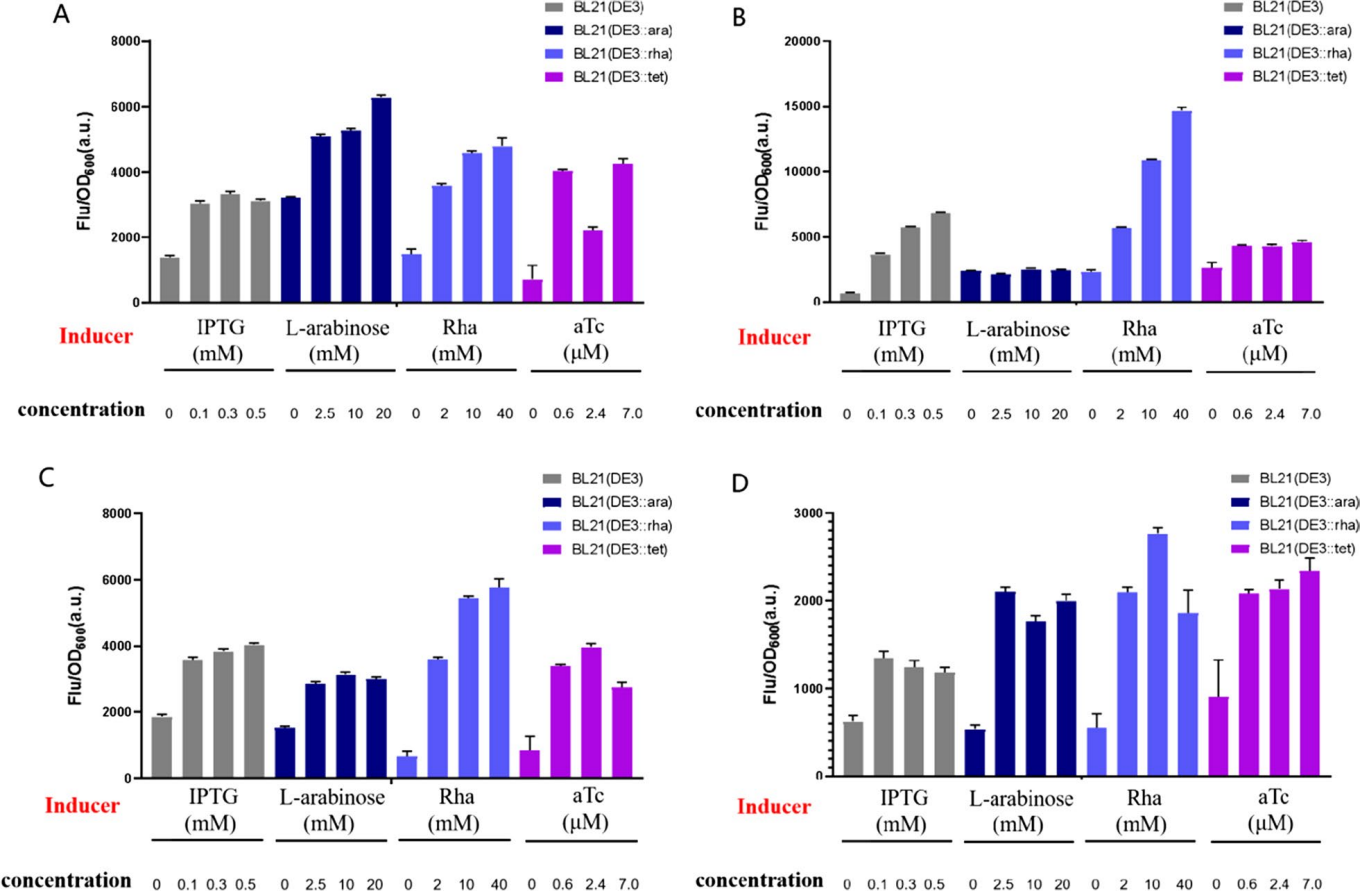
Tunable expression of GDH and three membrane proteins in three engineered strains and control strain BL21 (DE3) under different inducer concentrations at 36 h. **A** Tunable expression of GDH. **B** Tunable expression of Codb. **C** Tunable expression of Ecb. **D** Tunable expression of Yidc. Values and error bars represent the means and the deviations from triplicate experiments

The Fig. 4 showed the fluorescence intensity of four engineered strains producing different recombinant proteins mentioned above under different concentrations of inducers at 36 h. The fluorescence intensity of the three engineered strains were all higher than BL21 (DE3), when the two recombinant proteins GDH and Yidc were produced (Fig. 4a, d). Compared with BL21 (DE3), the unit cell fluorescence intensity of the three engineered strains were lower when the other two proteins were produced (Fig. 4b, c), which may be the result of the high cell biomass at the later stage of fermentation. It can be concluded from the results above that the concentration of inducer was closely related to protein expression. And the concentration of inducer required for optimal protein expression was different when producing different proteins. For example, the inducer with 20 mM l-arabinose had the best effect when GDH was expressed in BL21 (DE3::ara). For Codb and Ecb, when the strain BL21 (DE3::rha) was used as the production host, the required optimal Rha concentration was 40 mM. And Yidc protein production in strain BL21 (DE3::tet) was optimized when aTc concentration was 7.0 µM. In particular, proteins production in BL21 (DE3::rha) had a widest dynamic range and expression level when different concentration of rhamnose were added. And P_rhaBAD_ tunability is consistently good.

## Discussion

It is acknowledged that the pET expression system is a powerful tool for the production of recombinant proteins. BL21 (DE3) carries an inducible T7 RNA polymerase-dependent pET expression system that allows for the simple manipulation and tuning of protein production levels. However, there is still room for improvement of this gold-standard expression system [16]. For example, the original inducer’s chemical properties and the leaky expression of T7 RNAP does not favor recombinant proteins expression. IPTG is not an innocuous inducer, instead, it causes appreciable damage to the *E. coli* BL21 (DE3) host, which is already bearing a metabolic burden due to its content of plasmids carrying the expression genes [23]. Other studies had shown that this phenomenon was caused by too much T7 RNAP production sucks resources away from cellular growth and the leakage expression of T7 RNAP in the absence of inducer, which was toxic to cells when producing toxic proteins or membrane proteins [24, 25]. Moreover, the pET system shows “all or none” inducibility that does not allow detailed regulation of expression. In recent years, regulating the expression of T7 RNAP is an effective strategy for improving the recombinant protein production. Wagner et al. revealed that mutations in the promoter governing expression of T7 RNAP were key to the improved membrane protein overexpression characteristics of the Walker strains by using a combination of proteomics and genetics [26, 27]. Therefore, T7 RNAP inhibitor T7 Lys was used to dampen T7 RNAP activity to improve membrane protein production [28]. In order to achieve rigorous expression of T7 RNAP and further improve the production of hard-to-express proteins, this study adjusted the transcription expression and leakage level of T7 RNAP by replacing the promoter controlling T7 RNAP. Inducible promoters, especially P_rhaBAD_, can suppress leakage expression under non-induced conditions and realize tunability. The fluorescence intensity of strain BL21 (DE3) decreased obviously, and 97% of cells did not contain expression plasmid when the recombinant protein mentioned above were produced after 24 h (Figs. 2b, 3b, d, f). On the contrary, the fluorescence intensity of three engineered strains continued to rise, 98% of the cells still existed stably and played a role even after 48 h, which proved that selecting suitable promoters can reduce the growth burden of host cells. Previous studies had shown that P_rhaBAD_ controlled T7 RNAP to be placed on pET24 plasmid for gene expression [18], but this had the disadvantage that the plasmid cannot exist stably in the cell and cause mutations in the expressed genes. In this study, we modified the promoter controlling T7 RNAP on BL21 (DE3) chromosome by CRISPR/Cas9 technology, which not only solved the above limitations, but also achieved stable expression of T7 RNAP and alleviated metabolic burden caused by carrying extra plasmids.

In addition, the addition of IPTG is harmful to the cells, and the survival rate of the strain BL21 (DE3) was greatly reduced after 24 h, which was also verified in the above data (Fig. 3b, d, f). It had been proved that the concentration of IPTG which was only less than 0.1mM was non-toxic to cells [29], but this concentration cannot meet the requirements of our usual fermentation experiments. The three inducible promoters selected in this article, the inducers such as l-arabinose [11, 30], rhamnose [18, 31], anhydrotetracycline [32] had all been studied to control gene expression. Chou et al. developed the l-arabinose-induced pET system in the strain JM109 (DE3), and compared with BL21 (DE3), the production of penicillin acylase (PAC) was significantly improved, which proved that l-arabinose was more effective as an inducer than IPTG [33]. There was a research to prove that induction with l-arabinose can serve as a substitution to combined induction with 1 mM IPTG [11]. mlacI was placed under the P_rhaBAD_, which can strictly control the target gene and do not affect the growth of the strain, and rhamnose-inducible promoter had been widely used in the production of membrane proteins and secreted proteins of *E. coli* [31]. Recently, a new gene regulation tool based on anhydrotetracycline-inducible promoter had been developed [34]. Anyhow, a large number of studies had proved that the inducer used in this paper is non-toxic or slightly toxic to cells. More importantly, IPTG inducer is expensive and other low-cost inducers (i.e., l-arabinose and rhamnose) can meet the requirement of sustainable development. Therefore, the promoter engineering in this study can alleviate the pressure of cell survival caused by external environment and reduce the economic cost of protein production.

## Conclusions

Tunable protein expression is crucial for synthetic and system biology. However, the BL21 (DE3) is not suitable for the expression of all proteins, such as membrane proteins or autolysable proteins. In this work, three BL21 (DE3)-derived variant strains were constructed, and were further used to improve the production of GDH and membrane proteins. Compared with P_lacUV5_, the basal leakage expression of the three inducible promoters was lower, which can reduce cytotoxicity and accurately regulate the expression of T7 RNAP. The strain BL21 (DE3) had the best production effect before 18 h. However, the fluorescence intensity of the three engineered strains were all higher than BL21 (DE3) after 24 h, and the average ratio of plasmid-carrying cells were about 98%. BL21 (DE3) has no dominance in producing membrane proteins for a long time. The production of GDH and membrane proteins were further improved by prolonging the fermentation time without affecting the survival rate of the engineered strains. We developed three robust and novel BL21 (DE3)-derived variant strains, which provided more host choices for recombinant proteins production.

## Supplementary Information

**Additional file 1: Table S1**: Strains and plasmids used in this work. **Table S2**. Primers used in this work. 
