# Supplementary material for: Regulating the T7 RNA polymerase expression in E. coli BL21 (DE3) to provide more host options for recombinant protein production
Source: Microb Cell Fact. 2021 Sep 26;20:189. doi: 10.1186/s12934-021-01680-6 (PMC8474846; doi:10.1186/s12934-021-01680-6)
Supplement: Supplementary file 1 — Additional file 1: Table S1: Strains and plasmids used in this work. Table S2. Primers used in this work. [file 12934_2021_1680_MOESM1_ESM.docx]

# Additional file 1

**Table S1 Strains and plasmids used in this work**

| Strains | Characteristics | References |
| --- | --- | --- |
| E.coli DH5α | F-,φ80 dlacZ ΔM15, Δ (lacZYA –argF) U169, deoR, recA1, endA1, hsdR17(rK-, mK+), phoA, supE44 , λ-, thi -1, gyrA96 , relA1 | TaKaRa |
| E.coli BL21 (DE3) | F–ompT hsdS (rB–mB–) dcm+Tetr galλ (DE3) endA Hte [argU proL Camr] [argU ileY leuW Strep/Specr] | Thermo Fisher Scientific |
| Plasmids |  |  |
| pCas | pCas consisted of cas9, lambda Red, sacB, and the sgRNA guiding the pMB1 replication of pTarget. | Addgene 73227 |
| pTarget-ara | Constitutive expression of sgRNA with arabinose promoter(Sequence 1) editing template DNA. | This study |
| pTarget-rha | Constitutive expression of sgRNA with rhamnose promoter (Sequence 2) editing template DNA. | This study |
| pTarget-tet | Constitutive expression of sgRNA with tetracycline promoter (Sequence 3)editing template DNA. | This study |
| pET24a-EGFP | The EGFP gene from Escherichia coli was constructed into pET-24a vector. | This study |
| pET24a-GDH-EGFP | The GDH gene from Bacillus subtilis (CN104830921A) was constructed into pET-24a-EGFP vector. | This study |
| pET24a-Ecb-EGFP | The Ecb gene was constructed into pET-24a-EGFP vector. | This study |
| pET24a-Codb-EGFP | The Codb gene was constructed into pET-24a-EGFP vector. | This study |
| pET24a-Yidc-EGFP | The Yidc gene was constructed into pET-24a-EGFP vector. | This study |

**Table S2 Primers used in this work. All primers are synthesized by Synbio-tech Co., Ltd., China.**

| Primer | Sequence (5´ to 3´) |
| --- | --- |
| cm-N20-F | AGACGGTGAGCTGGTGATATGTTTTAGAGCTAGAAATAGC |
| cm-N20-R | ATATCACCAGCTCACCGTCTACTAGTATTATACCTAGGAC |
| gRNA-cm-F | GAGCTGATACCGCTCGCCGCACCGCCGGATGGTTACCTGC |
| gRNA-cm-R | CTGCGCTCGGTCGTTCGGCTGCATCTACTCGTCGCGAACC |
| Ara-P-F | GTAGGCGGATCCAGATCCCGTTATGACAACTTGACGGCTA |
| Ara-P-R | TAGCTGTTTCCTGTGTGAAATTTTTATAACCTCCTTAGAG |
| Rha-P-F | GTAGGCGGATCCAGATCCCGGGGCATGGCGAATTAATCTT |
| Rha-P-R | TAGCTGTTTCCTGTGTGAAAGGTTGGACTCAAGACGATAG |
| Tet-P-F | GTAGGCGGATCCAGATCCCGATGTATATCTCCTTTAAAGT |
| Tet-P-R | AGCTGTTTCCTGTGTGAAACTGATGAATCCCCTAATGATT |
| Ara-G-F | CTCTAAGGAGGTTATAAAAATTTCACACAGGAAACAGCTA |
| Ara-G-R | TAGCCGTCAAGTTGTCATAACGGGATCTGGATCCGCCTAC |
| Rha-G-F | CTATCGTCTTGAGTCCAACCTTTCACACAGGAAACAGCTA |
| Rha-G-R | AAGATTAATTCGCCATGCCCCGGGATCTGGATCCGCCTAC |
| Tet-G-F | AATCATTAGGGGATTCATCAGTTTCACACAGGAAACAGCT |
| Tet-G-R | ACTTTAAAGGAGATATACATCGGGATCTGGATCCGCCTAC |
| EGFP-F | TTTAAGAAGGAGATATACATATGGTGAGCAAGGGCGAGGA |
| EGFP-R | CAGTGGTGGTGGTGGTGGTGTTACTTGTACAGCTCGTCCA |
| EGFP-gu-F | TGGACGAGCTGTACAAGTAAATGTATATCTCCTTCTTAAA |
| EGFP-gu-R | TCCTCGCCCTTGCTCACCATCACCACCACCACCACCACTG |
| EGFP-Ecb-F | TTTAAGAAGGAGATATACATGTGAATCTTAACGCAACAAT |
| EGFP-Ecb-R | TGCTCACCATGCTGCCGCCGCCGCCTTACAGTTCAGCGACAAGTT |
| EGFP-Ecb-gu-F | AACTTGTCGCTGAACTGTAAGGCGGCGGCGGCAGCATGGTGAGCA |
| EGFP-Ecb-gu-R | ATTGTTGCGTTAAGATTCACATGTATATCTCCTTCTTAAA |
| EGFP-GDH-F | TCGAGGGCGGCGGCGGCAGCATGGTGAGCAAGGGCGAGGA |
| EGFP-GDH-R | CAGTGGTGGTGGTGGTGGTGTTACTTGTACAGCTCGTCCA |
| EGFP-GDH-gu-F | TGGACGAGCTGTACAAGTAACACCACCACCACCACCACTG |
| EGFP-GDH-gu-R | CCTTTTAAATCCGGATACATATGTATATCTCCTTCTTAAA |
| EGFP-Yidc-F | TTTAAGAAGGAGATATACATATGGATTCGCAACGCAATCT |
| EGFP-Yidc-R | TGCTCACCCATGCTGCCGCCGCCGCCTCAGGATTTTTTCTTCTCGC |
| EGFP-Yidc-gu-F | GCGAGAAGAAAAAATCCTGAGGCGGCGGCGGCAGCATGGTGAGCA |
| EGFP-Yidc-gu-R | AGATTGCGTTGCGAATCCATATGTATATCTCCTTCTTAAA |
| EGFP-Codb-F | TTTAAGAAGGAGATATACATGTGTCGAAAGATAACAACTT |
| EGFP-Codb-R | CAGTGGTGGTGGTGGGGTGTTACTTGTACAGCTCGTCCA |
| EGFP-Codb-gu-F | TGGACGAGCTGTACAAGTAACACCACCACCACCACCACT |
| EGFP-Codb-gu-R | AAGTTGTTATCTTGCGACACATGTATATCTCCTTCTTAAA |
